# Osteoblast-Specific Deletion of *Pkd2* Leads to Low-Turnover Osteopenia and Reduced Bone Marrow Adiposity

**DOI:** 10.1371/journal.pone.0114198

**Published:** 2014-12-02

**Authors:** Zhousheng Xiao, Li Cao, Yingjuan Liang, Jinsong Huang, Amber Rath Stern, Mark Dallas, Mark Johnson, Leigh Darryl Quarles

**Affiliations:** 1 Department of Medicine, University of Tennessee Health Science Center, Memphis, Tennessee, 38165, United States of America; 2 Department of Oral Biology, School of Dentistry, University of Missouri-Kansas City, Kansas City, Missouri, 64108, United States of America; 3 Engineering Systems, Inc., Charlotte, North Carolina, 28227, United States of America; Georgia Regents University, United States of America

## Abstract

Polycystin-1 (Pkd1) interacts with polycystin-2 (Pkd2) to form an interdependent signaling complex. Selective deletion of *Pkd1* in the osteoblast lineage reciprocally regulates osteoblastogenesis and adipogenesis. The role of Pkd2 in skeletal development has not been defined. To this end, we conditionally inactivated *Pkd2* in mature osteoblasts by crossing *Osteocalcin* (*Oc*)-Cre;*Pkd2* ^+/null^ mice with floxed *Pkd2* (*Pkd2* ^flox/flox^) mice. *Oc*-Cre;*Pkd2* ^flox/null^ (*Pkd2* ^Oc-cKO^) mice exhibited decreased bone mineral density, trabecular bone volume, cortical thickness, mineral apposition rate and impaired biomechanical properties of bone. *Pkd2* deficiency resulted in diminished *Runt-related transcription factor 2* (*Runx2*) expressions in bone and impaired osteoblastic differentiation *ex vivo*. Expression of osteoblast-related genes, including, *Osteocalcin*, *Osteopontin*, *Bone sialoprotein* (*Bsp*), *Phosphate-regulating gene with homologies to endopeptidases on the X chromosome* (*Phex*), *Dentin matrix protein 1* (*Dmp1*), *Sclerostin* (*Sost*), and *Fibroblast growth factor 23* (*FGF23*) were reduced proportionate to the reduction of *Pkd2* gene dose in bone of *Oc*-Cre;*Pkd2* ^flox/+^ and *Oc*-Cre;*Pkd2* ^flox/null^ mice. Loss of *Pkd2* also resulted in diminished peroxisome proliferator-activated receptor γ (*PPARγ*) expression and reduced bone marrow fat *in vivo* and reduced adipogenesis in osteoblast culture *ex vivo*. Transcriptional co-activator with PDZ-binding motif (TAZ) and Yes-associated protein (YAP), reciprocally acting as co-activators and co-repressors of Runx2 and PPAR*γ*, were decreased in bone of *Oc*-Cre;*Pkd2* ^flox/null^ mice. Thus, Pkd1 and Pkd2 have coordinate effects on osteoblast differentiation and opposite effects on adipogenesis, suggesting that Pkd1 and Pkd2 signaling pathways can have independent effects on mesenchymal lineage commitment in bone.

## Introduction

*PKD1* encodes polycystin-1 (PC1), a transmembrane receptor-like protein, which links environmental clues to intracellular processes regulating cell growth and development. *PKD2* encodes polycystin-2 (PC2), a transient receptor potential channel. The PC1 binds to PC2 in a ratio of 1 to 3 through their respective C-terminal coiled-coiled domains to form the functional signaling polycystin complex in cell surface membranes [1]–[5]. Typically, the functions of PC1 and PC2 are interdependent and concordant, as evidenced by common Autosomal Dominant Polycystic Kidney Disease (ADPKD) phenotype caused by inactivation of either PKD1 or PKD2 [6], [7].

Although the functions of polycystins have largely been derived from the study of inactivating mutations of either *PKD1* or *PKD2* in the kidney, *Pkd1* and *Pkd2* are widely expressed in many tissues and cell types, including the osteoblast lineage in bone. Recent studies indicate that PC1 (Pkd1) and PC2 (Pkd2) also form a complex that co-localize to primary cilia in osteoblasts/osteocytes to create a “sensor” that regulates bone mass [8]. Osteoblast lineage specific deletion of *Pkd1* in mice establishes a direct role for PC1 in regulating both osteoblast development and transducing the bone response to mechanical loading [8]–[15]. Indeed, the selective genetic ablation of *Pkd1* in osteoblasts and osteocytes results in osteopenia that is caused by diminished osteoblast-mediated bone formation and increased bone marrow adipogenesis. Loss of *Pkd1* reciprocally decreases *Runx2* and increases expression of *PPARγ* transcription factors that direct the commitment of mesenchymal stem cells to the osteoblastic and adipocytic lineages, respectively. Calcium-dependent signal transduction pathways link Pkd1 to Runx2 expression, but the cellular mechanisms mediating the reciprocal regulation of PPARγ have not been defined. The phenotype in the bone specific *Pkd1*-deficient mice resembles age-related bone loss, suggesting that understanding the function of polycystins in bone may be important in understanding the pathogenesis and treatment of senile osteoporosis.

Whether loss of Pkd2 function results in a bone phenotype in mice similar to *Pkd1* deficiency has not be investigated. However, recent siRNA mediated knock-down of *Pkd2* in osteoblasts resulted in impaired osteoblasts differentiation *in vitro* [16]. In addition, data from a GWAS meta-analyses found that the *PKD2* SNP rs12511728 was significantly associated with femoral neck bone mineral density (BMD)[16] and mutations in *PKD2* are associated with abnormal shape of craniofacial bones in patients with ADPKD [17]. Global homozygous *Pkd2* ^null/null^ mice die early in utero [18], which precludes assessing the direct effects of Pkd2 on osteoblast function.

To define the osteoblast specific functions of Pkd2, we conditionally inactivated *Pkd2* in postnatal mature osteoblasts. We found that loss of Pkd2 suppressed both osteoblast-mediated bone formation and adipogenesis, leading to osteopenia and decreased bone marrow fat. Thus, Pkd1 and Pkd2 have concordant effects on osteoblastogenesis and opposite effects on adipogenesis, consistent with both overlapping and independent signaling functions in osteoblasts.

## Materials and Methods

### Animal breeding and genotyping

**Figure 1:**
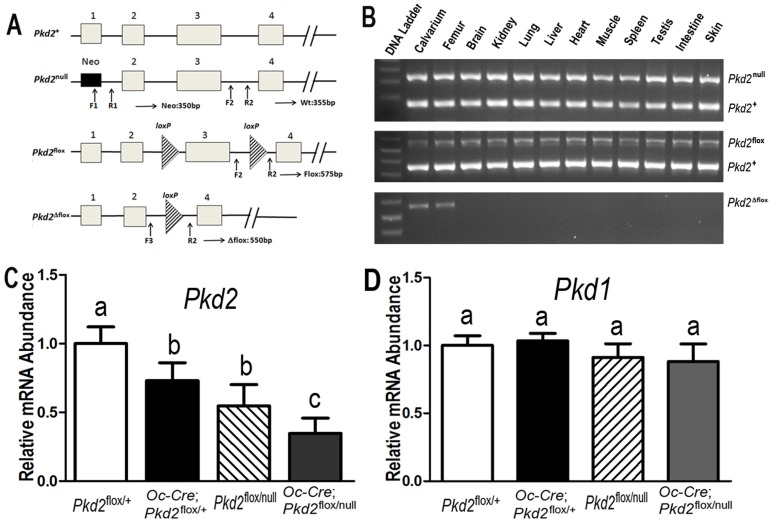
*Osteocalcin*(*Oc*)-Cre-mediated bone specific deletion of *Pkd2* from the floxed *Pkd2* allele (*Pkd2* ^flox^). (A) Schematic illustration of wild-type (*Pkd2* ^+^), null (*Pkd2* ^null^, deleted Exon 1), and floxed *Pkd2* allele before (*Pkd2* ^flox^) and after deletion (*Pkd2* ^Δflox^) of the lox P cassette containing Exon 3 via Cre-mediated recombination. (B) Genotype PCR analysis of different tissues that were harvested from 6-week-old *Oc*-Cre;*Pkd2* ^flox/null^ mice showed bone specific deletion of the *Pkd2* gene. *Osteocalcin*-Cre-mediated recombination of excised floxed *Pkd2* (*Pkd2* ^Δflox)^ allele occurred exclusively in bone, whereas non-skeletal tissues retained the floxed *Pkd2* allele (*Pkd2* ^flox^). (C and D) Real-time RT-PCR analysis of total *Pkd2* and *Pkd1* transcripts. Expression of total *Pkd2* transcripts was performed using *Pkd2-* or *Pkd1-*allele-specific primers as described in Experimental Procedures. The normal *Pkd2* ^+^ vs cyclophilin A is normalized to the mean ratio of 5 control mice, which has been set to 1. The percentage of conditional and global deleted transcripts was calculated from the relative levels of the normal *Pkd2* ^+^ transcripts in different *Pkd2* exons. Data are mean ±S.D. from 6–8 individual mice. Values sharing the same superscript in C and D are not significantly different at *P*<0.05.

All animal research was conducted according to guidelines provided by the National Institutes of Health and the Institute of Laboratory Animal Resources, National Research Council. The University of Tennessee Health Science Center's Animal Care and Use Committee approved all animal studies (Protocol number: 12–160.0). The mice were anesthetized with Ketamine (90 mg/kg) and Xylazine (10 mg/kg) for a bone densitometry scan, and the mice not useful for experimental purposes were sacrificed by CO_2_ inhalation plus cervical dislocation. We obtained the floxed *Pkd2* (in exon 3) mice and heterozygous *Pkd2* ^null/+^ (in exon 1) mice from Dr. Guanqing Wu at Vanderbilt University Medical Center [19] and *Osteocalcin* (*Oc*)-Cre mice from Dr. Thomas Clemens at University of Alabama [20]. These mice were bred and maintained on a C57BL/6J background. At first, we created double heterozygous *Oc*-Cre;*Pkd2* ^null/+^ mice and homozygous *Pkd2* ^flox/flox^ mice. Then double heterozygous *Oc*-Cre;*Pkd2* ^null/+^ mice were mated with homozygous *Pkd2* ^flox/flox^ mice to generate excised floxed *Pkd2* heterozygous (*Oc*-Cre;*Pkd2* ^flox/+^) and null mice (*Oc*-Cre;*Pkd2* ^flox/null^ or *Pkd2* ^Oc-cko^), as well as *Pkd2* heterozygous mice (*Pkd2* ^null/flox^) and *Oc*-Cre negative control mice (*Pkd2* ^flox/+^, equivalent to wild-type). These mice were used to collect samples at 6 weeks of age for phenotypic analysis. For genotyping PCR and Cre-mediated recombination, genomic DNAs were prepared from tail clips, bone, and other tissue specimens using a Tissue PCR Kit (Sigma-Aldrich, St. Louis, MO, USA). Mice were genotyped for *Oc-*Cre using previously described primers [12], for the *Pkd2^flox^* allele using forward primer 5′-TCT GAC TTG CAG ACT GTG GG-3′ and reverse primer 5′-AGG TAG GGG AAG GTC AGG GTT GG-3′ (355 bp product for the *Pkd2* ^+^ wild-type allele, 575 bp product for the *Pkd2^flox^* floxed allele), for the *Pkd2* ^Δflox^ delta floxed allele using forward primer 5′-AGC TTG GCT GGA CGT AAA-3′ and reverse primer 5′- AGG TAG GGG AAG GTC AGG GTT GG-3′ (427 bp product for the *Pkd2* ^Δflox^ delta floxed allele), and for the *Pkd2* ^null^ allele using two forward primers 5′-GCG CCG GCC TAG CTG TCC C-3′ and 5′-GTG CTA CTT CCA TTT GTC ACG TCC TGC-3′ and one reverse primer 5′-GTT GTC GCG GCT CCA CG-3′ (150 bp product for the *Pkd2* ^+^ wild-type allele, 350 bp product for the *Pkd2* ^null^ null allele) as previously described [19]. All *Pkd2* alleles were identified in 2% agarose gels (Figure 1).

### Bone densitometry, histomorphometric and micro-CT analysis

Bone mineral density (BMD) of femurs was assessed at 6 weeks of age using a small animal bone densitometer (Lunar Corp, Madison, WI). Calcein (Sigma-Aldrich, St. Louise, MO) double labeling of bone and histomorphometric analyses of periosteal mineral apposition rate (MAR) in tibias were performed using the osteomeasure analysis system (Osteometrics, Inc., Decatur, GA). The distal femoral metaphyses were also scanned using a micro-CT 40 scanner (Scanco Medical AG, Brüttisellen, Switzerland). A 3D images analysis was done to determine bone volume (BV/TV) and cortical thickness (Ct.Th) as previously described [12], [21].

### Three-point bending to failure

Femurs were harvested from freshly euthanized mice. The femurs (1520 slices per femur) were scanned with a micro-CT 40 scanner (Scanco Medical AG, Brüttisellen, Switzerland), and saved in a DICOM file format. To characterize the biomechanical properties of the bones, displacement-controlled three-point bending to failure tests were conducted on excised femurs *ex vivo*. For each femur, all associated soft tissues were removed; the bone was individually wrapped in saline-soaked gauze, and then stored at −20°C until testing. On the day of testing, samples were removed from −20°C storage, and allowed to thaw and reach room temperature. Prior to, and throughout the tests, bone samples were kept hydrated with saline soaked gauze and not allowed to dry out. To allow unbiased comparison between the samples, consistent span lengths of 6mm and crosshead displacement rates of 0.1 mm/s were used during testing. The femurs were placed into the test fixtures so they would be impacted in what would be the anterior to posterior direction *in vivo* (ElectroForce 3200, Bose Corp., Minnetonka, MN). Crosshead displacement and axial load were recorded at a rate of 70 Hz. The stiffness and ultimate force were calculated from the resulting load versus displacement curves for each sample. The Young's modulus (E) for each bone was calculated using the following equation:
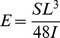
Where *S* is the stiffness, *L* is the span length, and *I* is the area moment of inertia. The area moment of inertia was calculated across the midspan at the fracture location using 10 slices midspan of the realigned microCT scans using the BoneJ plugin for the image-processing program ImageJ (Bone J, NIH, Bethesda, MD, USA) [8], [22], [23].

### Bone microindentation testing (BMT)

The BMT was performed using a microindentation device (ActiveLife Tech, Inc., Santa Barbara, CA, USA) as previously described [24]–[26]. Briefly, the periosteum of isolated femurs was scratched and a probe assembly placed on the anterior surface of the mid-femur performed measurements. A 10-cycle indentation with a maximum 2N force and 2 Hz frequency at a touchdown force of 0.1N and without any preconditioning was performed and the average value of three measurements was recorded. The indentation distances were analyzed by specific software and four parameters were obtained to use as outcome variables: indentation distance increase (IDI) between the first and last indentation cycle; total distance between the bone surface and the last indentation cycle (Total ID); creep indentation distance (Creep ID), the progressive indentation distance during the stable force phase of the first indentation cycle at the maximum 2N force; and average unloading slope, the average slope of unloading portion during three measurements. IDI values were then normalized by the IDI of polymethylmethacrylate (PMMA) tested with the same probe. Throughout testing samples were kept moist with 1xPBS.

### Detection of bone marrow adipocytes in long bones by oil red O lipid staining and micro-CT analysis

Whole intact femurs and tibias with encapsulated marrow were dissected from 20 weeks old mice, fixed for 48 hours in phosphate buffered paraformaldehyde, decalcified in 14% EDTA for two weeks. For oil red O lipid staining, the tibias were embedded in tissue freezing medium. Cryosectioning was performed on a frozen sectioning device (Leica, Nussloch, Germany) equipped with a rapid frozen sectioning kit (Instrumedics, Hackensack, NJ). 10 µm thick sections were then stained with oil red O (ORO) for bone marrow adipocytes as previously described [9], [12]. Briefly, the sections were rinsed in 60% isopropanol, stained for 20 minutes in 0.5% ORO-isopropanol solution, differentiated in 60% isopropanol, rinsed in tap water and mounted in glycerin jelly. Sections were examined with an inverted fluorescent microscope equipped with a digital camera (Leica, Nussloch, Germany). For detection of bone marrow fat cells by Micro-CT, the femurs were stained for 2 hours in 2% aqueous osmium tetroxide (OsO4). The Bones were rinsed in water for 48 hours and then scanned at 6 µm resolution using a micro-CT 40 scanner, 45 KeVp and 177 µA. Quantification of fat volume, density, and distribution throughout the marrow was registered to low contrast decalcified bone as our Lab previously described [9], [12].

### Real-time RT-PCR

For quantitative real-time RT−PCR, 1.0 µg total RNA isolated from either the long bone of 6-week-old mice or 10-days cultured primary osteoblasts in differentiation media was reverse transcribed as previously described [9], [27]. PCR reactions contained 100 ηg template (cDNA or RNA), 300 ηM each forward and reverse primers, and 1XqPCR Supermix (Bio-Rad, Hercules, CA) in 50 µl. The threshold cycle (Ct) of tested-gene product from the indicated genotype was normalized to the Ct for cyclophilin A. Expression of total *Pkd2* transcripts was performed using the following *Pkd2-*allele-specific primers: In exon 3, forward primer of normal *Pkd2* ^+^ transcript: 5′-GCA TGA TGA GCT CCA ATG TG-3′, and reverse primer: 5′-TCG ACA CTG GGG TGT CTA TG -3′. In exon 1, forward primer of normal *Pkd2* ^+^ transcript: 5′-TTG AGG CAG AGG AGG ATG AC-3′, and reverse primer: 5′-CAT CCA TCT CTA CCA CCA TCC-3′. The normal *Pkd2* ^+^ vs cyclophilin A is normalized to the mean ratio of 5 control mice, which has been set to 1. The percentage of conditional and global deleted transcripts was calculated from the relative levels of the normal *Pkd2* ^+^ transcripts in different *Pkd2* exons.

### Serum biochemistry

Serum urea nitrogen (BUN) was determined using a BUN diagnostic kit from Pointe Scientific, Inc. Calcium was measured using a calcium cresolphthalein complexone kit (Stanbio Laboratories, Boerne, TX, USA) and phosphorus was measured using the phosphomolybdylate-ascorbic acid method, as previously described [9], [11], [28]. Serum Osteocalcin levels were measured using a mouse Osteocalcin EIA kit (Biomedical Technologies Inc. Stoughton, MA, USA). Serum parathyroid hormone (PTH) levels were measured using the Mouse Intact PTH ELISA kit (Immutopics, Carlsbad, CA, USA). Serum full-length FGF23 levels were measured using the FGF23 ELISA kit (Kainos Laboratories, Tokyo, Japan). Serum OPG and Rank ligand (RankL) were measured using mouse ELISA kits (Quantikine, R&D Systems, Minneapolis, MN, USA), and serum tartrate resistant acid phosphatase (TRAP) was assayed with a mouse TRAP ELISA kit (Immunodiagnostic Systems, Fountain Hills, AZ).

### Primary osteoblast culture for proliferation, differentiation, and western blot analysis

Calvaria from E17.5 control and *Pkd2*-deficient embryos were used to isolate primary osteoblasts by sequential collagenase digestion at 37°C. To engineer immortal osteoblast cell lines, isolated primary osteoblasts were infected using a retroviral vector carrying SV40 large and small T antigen as previously described [10], [29]. Briefly, cells were grown in 100-mm plates at 50–60% confluence the day before infection. On the day of infection, the medium was removed and replaced with medium containing SV40 large and small T antigen-helper-free viral supernatant in the presence of 4 mg/ml of polybrene (Sigma, St. Louis, MO, USA) for 48 h. The cells were allowed to recover for 72 h followed by selection with 1 mg/ml puromycin (Sigma-Aldrich) for up to 15 days. The immortalized osteoblasts were cultured in α-MEM containing 10% FBS and 1% penicillin and streptomycin (P/S) and characterized following the protocols below. Cell proliferation was detected by BrdU incorporation assays following the manufacturer's directions (QIA58, Calbiochem, Gibbstown, NJ, USA). To induce differentiation, the immortalized osteoblasts were plated at a density of 2×10^4^ cells per well in a 12-well plate and 4×10^4^ cells per well in a 6-well plate and grown up to 21 days in α-MEM containing 10% FBS supplemented with 5 mM β-glycerophosphate and 25 µg/ml ascorbic acid. ALP activity and Alizarin red-S histochemical staining for mineralization were performed as previously described [10], [29]. Total DNA content was measured with a double-strand DNA quantitation reagent and kit (Molecular Probes, Eugene, OR, USA). Protein concentrations of the supernatant were determined with a total protein assay kit (Bio-Rad, Hercules, CA, USA). For signaling mechanism study, the cells were cultured in the osteogenic differentiation media for 48 hours. Then the cells were lysed with 150 µl of tissue protein extraction reagent (Pierce Biotechnology, Rockford, IL, USA) with 1 x Halt protease inhibitor and 1 mM phenylmethanesulfonyl fluoride (PMSF) per well, After three 30-second sonications, total cell lysates were centrifuged at 13,000×*g* for 10 minutes and supernatants were stored at -80°C until use. Protein concentrations of the supernatant were determined with a total protein assay kit (Bio-Rad, Hercules, CA). Equal quantities of protein were subjected to 4–12% Bis-Tris gradient Gels (Invitrogen, Carlsbad, CA) and were analyzed with standard western blot protocols (HRP-conjugated secondary antibodies from Santa Cruz Biotechnology and ECL chemiluminescent immunodetection system from GE Healthcare Bio-Sciences). Anti-YAP/TAZ (D24E4, #8418) and Anti-pYAP antibody (Ser127) (D9W2I, #13008) were purchased from Cell Signaling Technologies (Danvers, MA).Purified mouse anti-TAZ (560236) was purchased from BD Biosciences (San Jose, CA). Anti-p-TAZ (Ser 89) (sc-17610) and Anti-β-actin (sc-47778) antibodies were from Santa Cruz Biotechnology (Paso Robles, CA). The intensity of bands was quantified using Image J software (http://rsb.info.nih.gov/ij/).

### Transient transfection

The immortalized osteoblasts were cultured in α-MEM containing 10% fetal bovine serum (FBS) and 1% penicillin/streptomycin (P/S). To examine if PC2 regulates Hippo signaling pathway, a number of 1×10^6^ of the immortalized osteoblasts were transfected with 3.0 µg of 8xGTIIc luciferase reporter (8xGTIIc-Luc) constructs in combination with 3.0 µg of empty pcDNA3.1 expression vector, and 0.6 µg of Renilla luciferase-null (RL-null) as internal control plasmid by electroporation using a cell line optimal transfection kit according to the manufacturer's protocol (Amaxa Inc, Gaithersburg, MD). A total of 6.6 µg of plasmid DNAs was used for each electroporation. The transfected cells were plated in 12-well plates and harvested in 32 hours after transfection. Cells were lysed in 100 µl of reporter lysis buffer (Promega, Madison, WI). A luciferase assay (20 µl of cell lysed) was performed using a dual luciferase assay kit (Promega, Madison, WI), and activity was measured with a tube luminometer (MGM Instruments, Inc., Hamden, CT).

### Statistics

We evaluated differences between groups by one-way analysis of variance. All values are expressed as means ± S.D. All computations were performed using a commercial biostatistics software (GraphPad Software Inc. La Jolla, CA).

## Results

### *Oc*-Cre mediated bone-specific deletion of *Pkd2*

We studied four genotypes from the breeding strategy, including *Oc*-Cre;*Pkd2* ^flox/null^ or *Pkd2* ^Oc-cKO^, *Oc*-Cre;*Pkd2* ^flox/+^, *Pkd2* ^flox/null^, and *Pkd2* ^flox/+^. These mice were born at the expected Mendelian frequency and all genotypes exhibited survival indistinguishable from wild-type mice over the period of study. *Oc*-Cre expression is limited to cells of the osteoblast lineage (late osteoblasts>osteocytes) with onset of expression just before birth [20]. To confirm that the *Pkd2* floxed allele in Exon 3 was selectively deleted in bone, we performed PCR analysis using a combination of primers that specifically detect floxed *Pkd2* alleles (*Pkd2* ^flox^) and the excised floxed *Pkd2* alleles (*Pkd2* ^Δflox^) in *Oc*-Cre;*Pkd2* ^flox/+^ or *Oc*-Cre;*Pkd1* ^flox/null^ mice (Figure 1A). We demonstrated that *Oc*-Cre-mediated floxed recombination occurred exclusively in bone, whereas non-skeletal tissues retained the intact floxed *Pkd2* alleles (*Pkd2* ^flox^) (Figure 1B). The *Pkd2* ^null^ null allele in Exon 1 was used in combination with the Cre-recombinase deletion of the floxed *Pkd2* allele (*Pkd2* ^Δflox^) in Exon 3 to increase the net efficiency of *Pkd2* inactivation.

The level of the full-length *Pkd2* transcripts in Exon 1 and 3 from the femurs of these four genotypes mice were assessed by real time RT-PCR. As expected, both *Pkd2* ^flox/null^ and *Oc*-Cre;*Pkd2* ^flox/null^ mice displayed 50% reduction of intact *Pkd2* transcripts in Exon 1, whereas *Oc*-Cre;*Pkd2* ^flox/+^ and *Oc*-Cre;*Pkd2* ^flox/null^ mice exhibited approximately 25% excision of the floxed Exon 3 from *Pkd2*, indicating that *Oc*-Cre mediated bone-specific deletion of the floxed *Pkd2* allele is incomplete (Figure 1C). The combined effect *Pkd2* ^null^ and (*Pkd2* ^Δflox^) in *Oc*-Cre;*Pkd2* ^flox/null^, however, resulted in a net reduction of intact *Pkd2* expression by ∼75% in bone (Figure 1C). Thus, there was a progressive reduction of functional *Pkd2* message in these *Pkd2* deficient mice, *i.e.*, *Pkd2* ^flox/+^ (100%), *Oc*-Cre;*Pkd2* ^flox/+^ (75%), *Pkd2* ^flox/null^ (50%), and *Oc*-Cre;*Pkd2* ^flox/null^ (25%) mice (Figure 1C). In contrast, targeting *Pkd2* had no effect on *Pkd1* transcript expression (Figure 1D). In addition, *Oc*-Cre;*Pkd2* ^flox/null^, and *Oc*-Cre;*Pkd2* ^flox/+^ demonstrated no cyst formation in the kidney, consistent with the bone specific inactivation of *Pkd2* (data not shown). *Pkd2* ^flox/null^ mice also exhibited no renal cyts, as previously reported [18].

### Effects of bone specific deletion of *Pkd2* on bone structure

**Figure 2:**
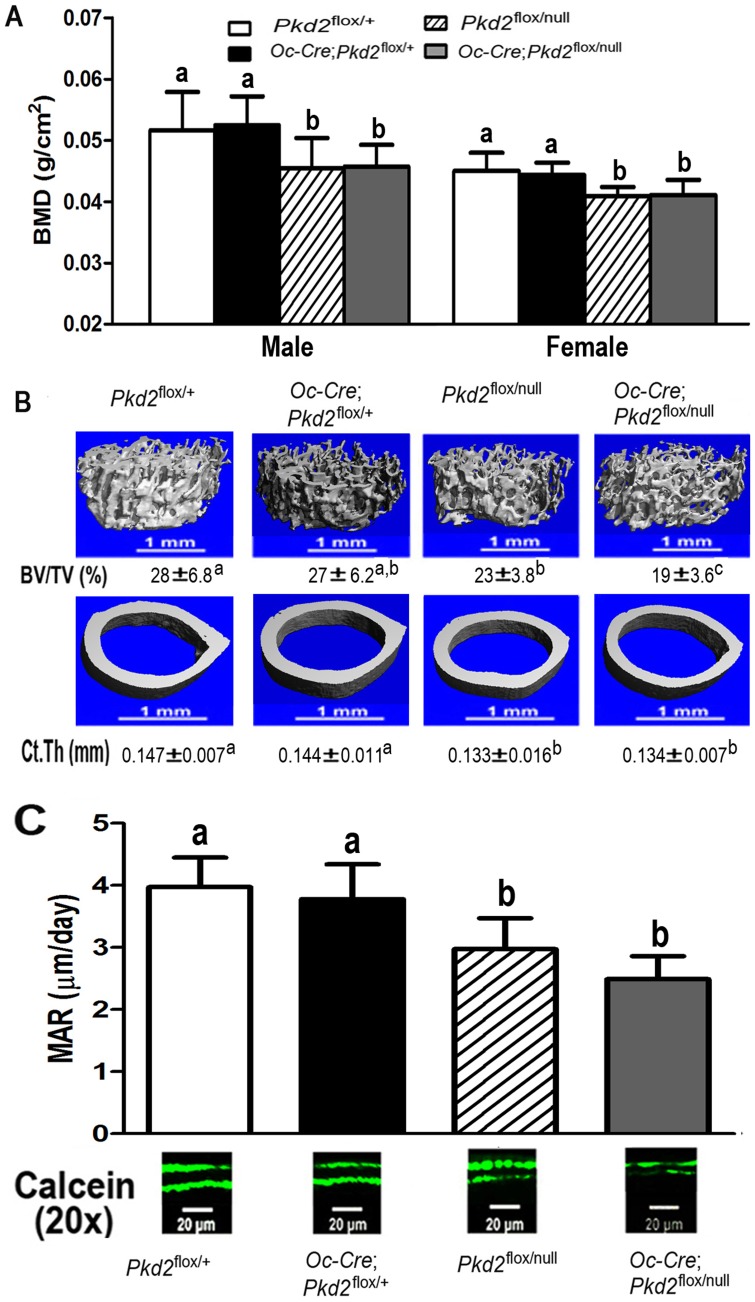
*Pkd2* deficiency results in loss of bone mass. (A) Effects of *Pkd2* deficiency on bone mineral density (BMD) at 6 weeks of age. Compared to control *Pkd2* ^flox/+^ mice, conditional heterozygous *Oc*-Cre;*Pkd2* ^flox/+^ mice exhibited no abnormalities in bone mass. However, both global heterozygous *Pkd2* ^flox/null^ and conditional *Pkd2* ^Oc-cKO^ null mice were osteopenic, as evidenced by respective 14.4% and 12.5% reduction BMD in male adult mice. (B) Effects of *Pkd2* deficiency on structure of femurs. µCT analysis of the distal femoral metaphyses and midshaft diaphyses revealed that the reduction in bone mass in both global heterozygous *Pkd2* ^flox/null^ and conditional *Pkd2* ^Oc-cKO^ null mice were caused by reductions in trabecular bone volume (19.3% and 30.9%, respectively) and cortical bone thickness (8.4% and 7.3%, respectively). In contrast, conditional heterozygous *Oc*-Cre;*Pkd2* ^flox/+^ had no significant difference in both trabecular and cortical bone compared with control mice. (C) Effects of *Pkd2* deficiency on bone formation rate. Both *Pkd2* ^flox/null^ and *Pkd2* ^Oc-cKO^ null mice exhibited a significant decrease in periosteal mineral apposition rate (MAR, 25.1% and 37.3%, respectively), indicating a reduction of bone formation rate in *Pkd2* deficient mice. Data represent the mean±S.D. from 6–8 individual mice. Values sharing the same superscript in different group are not significantly different at *P*<0.05.

At 6 weeks of age, the gross appearance and body weight of all genotypes were indistinguishable. Compared to control *Pkd2* ^flox/+^ mice, conditional heterozygous *Oc*-Cre;*Pkd2* ^flox/+^ mice, which had a 25% reduction in *Pkd2* expression, exhibited no abnormalities in bone mass. However, both global heterozygous *Pkd2* ^flox/null^ and conditional *Pkd2* ^Oc-cKO^ null mice, which had respective 50% and 75% reductions in *Pkd2* express, were osteopenic, as evidenced by respective 14.4% verse 9.2% and 12.5% verse 9.2% reductions in BMD in both male and female adult mice (Figure 2A). Micro-CT analysis revealed that the reduction in bone mass in both male global heterozygous *Pkd2* ^flox/null^ and conditional *Pkd2* ^Oc-cKO^ null mice were caused by reductions in trabecular bone volume (19.3% and 30.9%, respectively) and cortical bone thickness (8.4% and 7.3%, respectively) (Figure 2B). In contrast, conditional heterozygous *Oc*-Cre;*Pkd2* ^flox/+^ had no significant difference in both trabecular and cortical bone compared with control mice (Figure 2B). Consistent with a low-bone-mass phenotype by BMD and Micro-CT analysis, we found that loss of *Pkd2* in bone from both *Pkd2* ^flox/null^ and *Pkd2* ^Oc-cKO^ null mice was associated with a decrease in periosteal mineral apposition rate (MAR, 25.1% and 37.3%, respectively) (Figure 2C), indicating a significant reduction of bone formation rate in *Pkd2* deficient mice.

### Effect of conditional deletion of *Pkd2* on femur cortical bone geometry, mechanical and material properties

Conditional deletion of *Pkd2* also resulted in site-specific alterations of femur cortical bone geometry. In this regard, single conditional *Oc*-Cre;*Pkd2* ^flox/+^ heterozygous, single global *Pkd2* ^flox/null^ heterozygous, and conditional *Pkd2* ^Oc-cKO^ null mice showed a gene dose dependent decrease in both total and cortical area at 6 weeks of age when compared with age-matched control *Pkd2* ^flox/+^ mice (Table 1), this is in accordance with a significant reduction in moment of inertia, *I_x_* in these *Pkd2* deficient mice (Table 1). Obviously, *Pkd2* deficient mice displayed a reduction in the size of the marrow cavity resulting from a smaller midshaft diameter compared with control mice. To examine whether changes of femoral bone geometry in the *Pkd2* deficient mice may affect bone mechanical properties, we used these femurs to perform 3-point bending to failure experiments. Compared with the control mice, global *Pkd2* ^flox/null^ heterozygous and conditional *Pkd2* ^Oc-cKO^ null mice exhibited a lower maximum force and stiffness in 3-point bending, but no significant differences in the Young's modulus (Table 1), indicating that the changes of bone geometry and bone structure at 6 weeks of age may preserve the Young's modulus in global *Pkd2* ^flox/null^ heterozygous and conditional *Pkd2* ^Oc-cKO^ null mice. There was no difference in these parameters between single conditional *Oc*-Cre;*Pkd2* ^flox/+^ heterozygous mice and control mice (Table 1).

**Table 1:** Femoral bone geometry, biomechanical, and biomaterial properties in 6-week-old mice.

Parameters	*Pkd2* ^flox/+^	*Oc-Cre;Pkd2* ^flox/+^	*Pkd2* ^flox/null^	*Pkd2* ^Oc-cKO^	*p-value*
***Cortical bone Geometry***					
Total area (mm^2^)	2.5±0.19^a^	2.3±0.17^b^	2.1±0.27^c^	2.1±0.20^c^	<0.0001
Cortical area (mm^2^)	0.61±0.06^a^	0.56±0.06^a,b^	0.52±0.09^b^	0.52±0.05^b^	0.0017
Moment of inertia, *I*_x_ (mm^4^)	0.15±0.04^a^	0.11±0.03^b^	0.10±0.02^b^	0.11±0.03^b^	0.0007
***Biomechanical properties***					
Maximum force (N)	12.0±0.97^a^	10.8±1.2^a,b^	8.9±0.94^b^	9.4±1.6^b^	0.0077
Stiffness (N/mm)	60.2±9.1^a^	62.0±3.4^a^	46.9±5.6^b^	37.1±8.3^b^	0.0001
Young's modulus (Gpa)	2.9±1.0^a^	2.9±0.59^a^	3.4±0.52^a^	2.9±0.72^a^	0.5743
***Biomaterial properties***					
IDI (µm)	7.7±1.6^a^	9.7±2.2^a^	25.1±3.2^b^	22.3±5.3^b^	0.0053
Total ID (µm)	39.4±4.5^a^	47.6±10.6^a^	71.9±7.1^b^	66.7±7.7^b^	0.0015
Creep ID (µm)	4.5±0.84^a^	5.3±0.82^a^	11.8±1.3^b^	9.8±3.2^b^	0.0003
Average US (N/µm)	0.18±0.04^a^	0.18±0.02^a^	0.12±0.03^b^	0.12±0.02^b^	0.0077

Data are mean ±S.D. from 6-8 serum samples of 6-week-old individual mice. Values sharing the same superscript are not significantly different at *P*<0.05.

To assess whether *Pkd2* deficiency may have an impact on bone material properties, we used these femurs to perform reference point indentation (RPI, micro-indentation) measurements. We found significant differences in several RPI variables (Table 1) between *Pkd2* deficient and control mice. There was a 226%, 82.5%, and 162% increase in IDI, TID, and CID, respectively, between heterozygous *Pkd2* ^flox/null^ and control mice. A similar 190%, 69%, and 118% increase in IDI, TID, and CID, respectively, was also observed between *Pkd2* ^Oc-cKO^ null and control mice. In contrast, there was a 33.3% decrease in the average US in both global *Pkd2* ^flox/null^ heterozygous and conditional *Pkd2* ^Oc-cKO^ null mice compared with control mice. However, these parameters were not significantly different between heterozygous *Oc*-Cre;*Pkd2* ^flox/+^ and control mice (Table 1). These findings that *Pkd2* deficiency makes bone more brittle and less stiff.

### Pkd2-regulated gene expression in bone

Next, we examined by real-time RT-PCR the expression levels of a panel of osteoblast lineage-, osteoclast-, chondrocyte-, and adipocyte-related mRNAs from the femurs of 6-week-old control, heterozygous *Pkd2* deficient (*Oc*-Cre;*Pkd2* ^flox/+^ and *Pkd2* ^flox/null^), and *Oc*-Cre;*Pkd2* ^flox/null^ mice (Table 2). We found that changes in bone transcripts were more sensitive to reductions in *Pkd2* and exhibited a gene-dose dependent response on osteoblast-related gene transcripts.

**Table 2:** Gene-expression profiles in bone in 6-week-old mice.

Gene	Accession no.	*Oc-Cre;Pkd2* ^flox/+^	*Pkd2* ^flox/null^	*Oc-Cre; Pkd2* ^flox/null^	*p*-value
***Osteoblast lineage***					
*Runx2-II*	NM_009820.5	1.03±0.23	0.64±0.14*^,#^	0.46±0.28*^,#^	0.0005
*Osteopontin*	NM_009263.3	0.79±0.19*	0.71±0.15*	0.45±0.15*^,#,&^	<0.0001
*Osteocalcin*	NM_007541.2	0.76±0.16*	0.77±0.16*	0.51±0.13*^,#,&^	<0.0001
Bsp	NM_008318.3	0.87±0.12	0.68± 0.12*	0.37±0.24*^,#^	<0.0001
*Cox2*	NM_011198.3	0.76±0.18*	0.77±0.16*	0.50±0.15*^,#,&^	0.0001
*Cnx43*	NM_010288.3	0.93±0.23	0.95±0.18	0.41±0.18*^,#,&^	<0.0001
Mmp13	NM_008607.2	0.87±0.31	0.66± 0.21*	0.44±0.18*^,#^	0.0004
*Opg*	NM_008764.3	0.98±0.24	0.87±0.31	0.84±0.27	0.5987
*RankL*	NM_011613.3	0.85±0.18	0.94±0.19	0.56±0.21*^,#,&^	0.0014
*Phex*	NM_011077.2	0.90±0.41	0.97±0.39	0.43±0.14*^,#,&^	0.0040
*Dmp1*	NM_016779.2	0.91±0.27	0.79±0.23	0.52±0.21*^,#,&^	0.0014
*Mepe*	NM_053172.2	1.10±0.40	0.72±0.12*^,#^	0.56±0.20*^,#^	0.0017
*Sost*	NM_024449.5	0.98±0.35	0.68±0.12*^,#^	0.58±0.18*^,#^	0.0012
*Fgf23*	NM_022657.4	0.92±0.33	0.88± 0.38	0.63±0.18*	0.0576
*Wnt10b*	NM_011718.2	0.92±0.42	0.71±0.10*	0.35±0.24*^,#,&^	<0.0001
*Axin2*	NM_015732.4	0.94±0.17	0.72±0.26*	0.38±0.13*^,#,&^	<0.0001
Taz	NM_1168281.1	0.75±0.22*	0.72± 0.16*	0.42±0.24*^,#,&^	0.0002
Yap	NM_009534.3	1.05±0.11	0.74±0.17*^,#^	0.53±0.34*^,#^	0.0003
*BirC3*	NM_007464.3	1.00±0.18	0.72±0.18*^,#^	0.65±0.18*^,#^	0.0006
*Ctgf*	NM_010217.2	0.97±0.33	0.66±0.18*^,#^	0.45±0.21*^,#^	<0.0001
Inhba	NM_008380.1	0.98±0.21	0.71±0.16*^,#^	0.69±0.11*^,#^	0.0002
Hprt	NM_013556.2	1.07±0.15	0.95±0.23	1.08±0.41	0.7324
**Osteoclast**					
*Trap*	NM_007388.3	0.79±0.28	0.85±0.29	0.46±0.21*^,#,&^	0.0005
*Mmp*9	NM_013599.3	0.91±0.32	0.99±0.31	0.56±0.14*^,#,&^	0.0032
**Chondrocyte**					
*Collagen II*	NM_031163.3	0.91±0.16	0.90±0.33	1.29±0.0.71	0.2935
*VegfA*	NM_009505.4	1.15±0.21	1.10±0.29	0.99±0.37	0.7891
**Adipocyte**					
*PPARγ*	NM_009505.4	1.03±0.32	0.70±0.15*^,#^	0.57±0.17*^,#^	0.0008
*aP2*	NM_024406.2	1.16±0.31	0.98±0.27	0.64±0.37*^,#,&^	0.0157
*Lpl*	NM_008509.2	0.77±0.22	0.58±0.27*	0.66±0.29*	0.0136

Data are mean ±S.D. from 5–6 tibias of 6-week-old individual mice and expressed as the fold changes relative to the housekeeping gene cyclophilin A subsequently normalized to control mice. * indicates significant difference from control *Pkd2* ^flox/+^ mice, ^#^ indicates significant difference from *Oc-Cre;Pkd2* ^flox/+^ mice, ^&^ indicates significant difference from *Pkd2* ^null/flox^ mice at *p<*0.05, respectively.

We found significant alterations in osteoblast-related gene expression. Bone derived from heterozygous *Oc*-Cre;*Pkd2* ^flox/+^, in spite of having no measurable change in bone structure and only a 25% reduction in *Pkd2* message expression, did have significant reductions in several osteoblast-lineage gene transcripts, including *Osteocalcin, Osteopontin*, and *Cyclooxygenase 2* (*Cox2*) mRNA levels compared to control mice. Bone from heterozygous *Pkd1* ^flox/null^ mice with a 50% reduction in *Pkd2* expression exhibited significant reductions in *Runx2*-II, *Osteocalcin, Osteopontin, Bsp, Cox2, matrix metalloproteinase 13* (*Mmp13*), *matrix extracellular phosphoglycoprotein* (*Mepe*), and *Sost* mRNA levels compared to control mice (Table 2). Compared with heterozygous *Pkd2* ^flox/null^ mice, significantly greater reductions of *Osteocalcin, Osteopontin, Cox2, Connexin 43* (*Cnx43*), *Rank ligand (RankL)*, *Phex, Dmp1* and *Fgf23* were observed in conditional *Pkd2* ^Oc-cKO^ null mice that had a 75% reduction in *Pkd2* expression.

Changes in gene expression in bone correlated with alterations in serum biomarkers in conditional *Pkd2* ^Oc-cKO^ null mice. In this regard, further evidence for osteoblast-lineage dysfunction includes reductions in both Fgf23 and RanKL in serum from conditional *Pkd2* ^Oc-cKO^ null mice at 6 weeks of age (Table 3). However, no changes of serum phosphorus and calcium levels were observed in *Pkd2* ^Oc-cKO^ null mice compared to control mice. Also, no significant changes of serum BUN, PTH, Osteocalcin, and OPG were observed among these four genotype mice (Table 3).

**Table 3:** Biochemistry analysis in 6-week-old mice.

Parameters	*Pkd2* ^flox/+^	*Oc-Cre;Pkd2* ^flox/+^	*Pkd2* ^flox/null^	*Pkd2* ^Oc-cKO^	*p-value*
BUN (mg/dl)	18±3.2^a^	19±3.8^a^	18±2.7^a^	17±2.3^a^	0.5972
FGF23 (pg/ml)	127±27^a^	125±23^a^	108±29^a^	73±36^b^	0.0066
PTH (pg/ml)	44±19^a^	54±26^a^	64±23^a^	32±15^a^	0.0714
P (mg/dl)	10.3±0.90^a^	11.0±1.01^a^	11.4±0.98^a^	11.1±0.67^a^	0.1229
Ca (mg/dl)	8.7±0.08^a^	8.7±0.11^a^	8.7±0.17^a^	8.7±0.15^a^	0.4528
Osteocalin (ng/dl)	124±104^a^	92±67^a^	122±41^a^	104±59^a^	0.7657
OPG (ng/ml)	3.9±0.71^a^	3.9±0.48^a^	3.5±0.95^a^	3.9±0.51^a^	0.5027
RanKL (pg/dl)	83±14^a^	81±21^a^	77±20^a^	54±17^b^	0.0346
TRAP (U/L)	19±1.8^a^	18±1.9^a^	19±3.7^a^	15±1.8^b^	0.0338
1,25(OH)_2_D (pg/ml)	59±8.1^a^	57±10.3^a^	56±5.4^a^	55±11.3^a^	0.8022

Data are mean ±S.D. from 6-8 serum samples of 6-week-old individual mice. Values sharing the same superscript are not significantly different at *P*<0.05.

The *Opg/RankL* expression ratio was increased in conditional *Pkd2* ^Oc-cKO^ null mice and was associated with reduced bone expression of *Trap* and matrix metalloproteinase 9 (*Mmp9*), markers of bone resorption (Table 2). Serum levels of TRAP were also reduced in conditional *Pkd2* ^Oc-cKO^ null mice compared to control littermates (Table 3), suggesting that *Pkd2*-mediated bone loss results from low bone formation rates rather than increased bone resorption. Transcripts of chondrocyte-related genes did not differ between heterozygous *Pkd2* deficient and *Pkd2* ^Oc-cKO^ null mice (Table 2).

**Figure 3:**
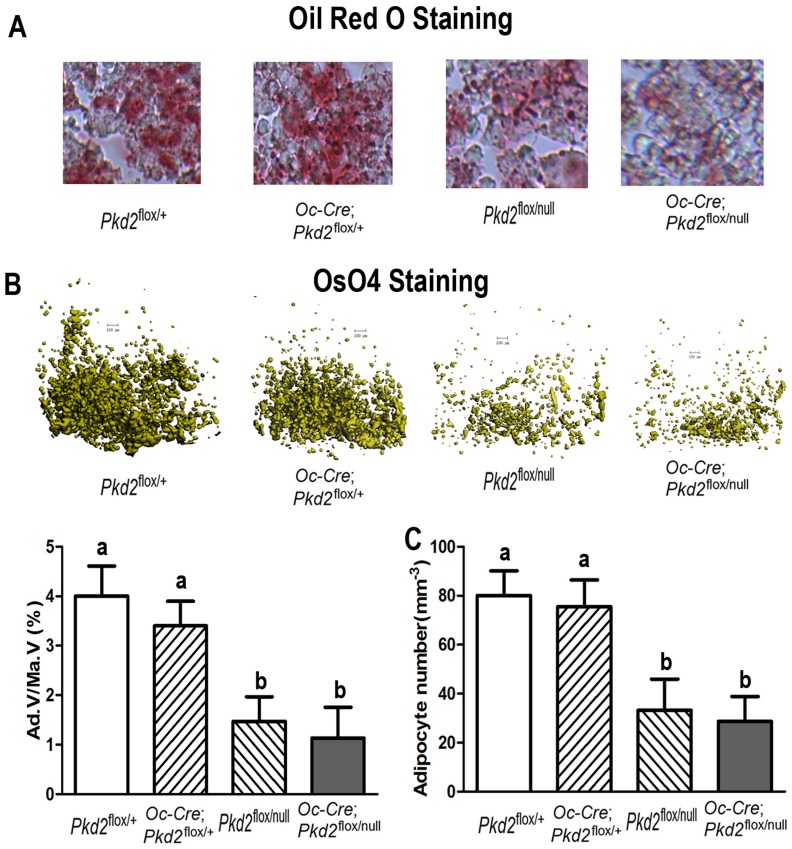
*Pkd2* deficiency impairs adipogenesis in bone. (A) Histology of adipocytes in decalcified tibias. Oil Red O staining showed that the numbers of adipocytes and fat droplets in tibial bone marrow were much less in 20-week-old *Pkd2* ^flox/null^ and *Pkd2* ^Oc-cKO^ null mice compared with age-matched control *Pkd2* ^flox/+^ and *Oc*-Cre;*Pkd2* ^flox/+^ mice. (B) Osmium tetroxide (OsO4) staining of decalcified femurs by µCT analyses. Upper panel showed the representative images of distal femoral bone marrow by OsO4 staining (yellow). Lower panel displayed adipocyte volume/marrow volume (Ad.V/Ma.V, %) and adipocyte number (Ad.N, mm^−3^) by calculation. Consistent with Oil Red O staining, µCT analyses showed that adipocyte volume/marrow volume (Ad.V/Ma.V, %) and adipocyte number (Ad.N, mm^−3^) were much lower in the distal femurs from 20-week-old *Pkd2* ^flox/null^ and *Pkd2* ^Oc-cKO^ null mice compared with age-matched control *Pkd2* ^flox/+^ and *Oc*-Cre;*Pkd2* ^flox/+^ mice, indicating an impairment of adipogenesis in the *Pkd2* deficient mice. Data represent the mean±S.D. from 6–8 individual mice. Values sharing the same superscript in different group are not significantly different at *P*<0.05.

*PPAR*γ, an adipocyte transcription factor, and adipocyte markers, including lipoprotein lipase (*Lpl*) and adipocyte fatty acid-binding protein 2 (*aP*2) were significantly decreased in femurs of in both heterozygous *Pkd2* ^flox/null^ and conditional *Pkd2* ^Oc-cKO^ null mice (Table 2). Consistent with attenuated adipogenic markers, bone marrow exhibited an decreased percentage of fat cells in both *Pkd2* ^flox/null^ and *Pkd2* ^Oc-cKO^ mice at 20 weeks of age, as evidenced by a lower number of adipocytes and fat droplets in decalcified femurs and tibias stained with Oil Red O (Figure 3A) and Osmium tetroxide (OsO4) (Figure 3B-3C), indicating an impairment of adipogenesis in *Pkd2* deficient mice. Thus, the effect of selective deletion of *Pkd2* in osteoblasts on adipogenesis is opposite to the effect of *Pkd1* deficiency.

### Effect of conditional deletion of *Pkd2* on osteoblastic function *ex vivo*

**Figure 4:**
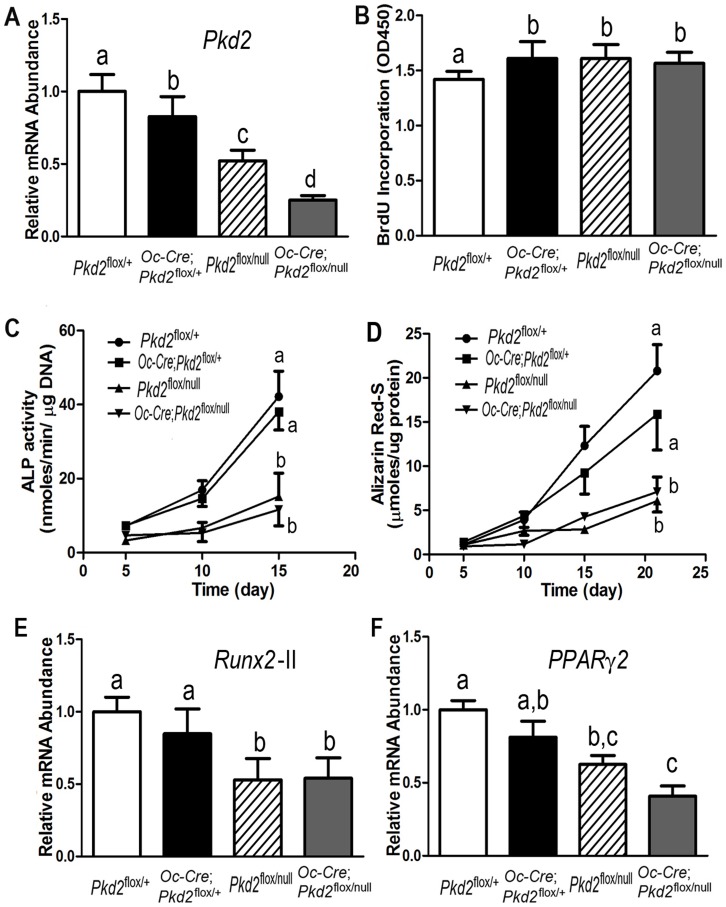
*Pkd2* deficiency osteoblasts have a developmental defect *in vitro*. (A) A real-time RT-PCR analysis of total *Pkd2* transcripts in osteoblast cultures. A gene dose-dependent reduction of *Pkd2* transcripts was observed in immortalized control and *Pkd2*-deficient osteiblasts. (B) BrdU incorporation. Primary cultured *Pkd2* deficient osteoblasts exhibited a higher BrdU incorporation than control *Pkd2* ^flox/+^ osteoblasts for 6 hours, indicating increased proliferation rate in the *Pkd2*-deficient osteoblasts. (C) ALP activity. Primary cultured *Pkd2* ^flox/null^ and *Pkd2* ^Oc-cKO^ null osteoblasts displayed time-dependent increments in alkaline phosphatase (ALP) activities during 15 days of culture, but the ALP activity was significantly lower at different time points compared with control *Pkd2* ^flox/+^ and *Oc*-Cre;*Pkd2* ^flox/+^ osteoblasts. (D) Quantification of mineralization. Alizarin Red-S was extracted with 10% cetylpyridinium chloride and quantified as described in Experimental Procedures. Primary cultured *Pkd2* ^flox/null^ and *Pkd2* ^Oc-cKO^ null had time-dependent increments in Alizarin Red-S accumulation during 22 days of culture, but the accumulation was significantly lower at different time points compared with control *Pkd2* ^flox/+^ and *Oc*-Cre;*Pkd2* ^flox/+^ osteoblasts. (E and F) Gene expression profiles by real-time RT-PCR. 10-days cultured *Pkd2* ^flox/null^ and *Pkd2* ^Oc-cKO^ null osteoblasts in osteogenic differentiation media showed a significant attenuation in both osteogenesis and adipogenesis compared to control *Pkd2* ^flox/+^ and *Oc*-Cre;*Pkd2* ^flox/+^, evidenced by a significant reduction in osteoblastic and adipogenic markers, such as *Runx2*-*II* and *PPARγ2*. Data are mean±S.D. from triple three independent experiments. Values sharing the same superscript in different group are not significantly different at *P*<0.05.

We confirmed that there was a gene dose-dependent reduction of *Pkd2* transcripts in osteoblasts derived from control *Pkd2* ^flox/+^, heterozygous *Oc*-Cre;*Pkd2* ^flox/+^ and *Pkd2* ^flox/null^, and *Pkd2* ^Oc-cKO^ mice (Figure 4A). To determine the impact of conditional deleted *Pkd2* on osteoblast function *ex vivo*, we examined cell proliferation and osteoblastic differentiation and gene expression profiles in immortalized primary osteoblast cultures derived from control *Pkd2* ^flox/+^, heterozygous *Oc*-Cre;*Pkd2* ^flox/+^ and *Pkd2* ^flox/null^, and *Pkd2* ^Oc-cKO^ null mice. Consistent with defects in bone formation, we found that *Pkd2* deficiency osteoblasts had a higher BrdU incorporation than control osteoblasts, indicating an increased proliferation rate in these *Pkd2* deficiency osteoblasts (Figure 4B). In addition, both heterozygous *Pkd2* ^flox/null^ and *Pkd1* ^Oc-cKO^ null osteoblasts displayed impaired osteoblastic differentiation and maturation, as evidenced by lower alkaline phosphatase activity, diminished calcium deposition in extracellular matrix, and reduced osteoblastic differentiation markers such as *Runx2*-II compared to controls (Figure 4C-4E). In agreement with decreased adipogenic activity *in vivo*, the cultured immortalized primary osteoblasts under osteogenic condition exhibited a markedly decrease of adipocyte markers such as *PPARγ2* in both heterozygous *Pkd2* ^flox/null^ and *Pkd1* ^Oc-cKO^ null osteoblast cultures (Figure 4F), suggesting an impairment of both osteogenesis and adipogenesis in the *Pkd2* deficient osteoblasts.

### Effects of *Pkd2* deficiency on signaling pathways in osteoblasts

**Figure 5:**
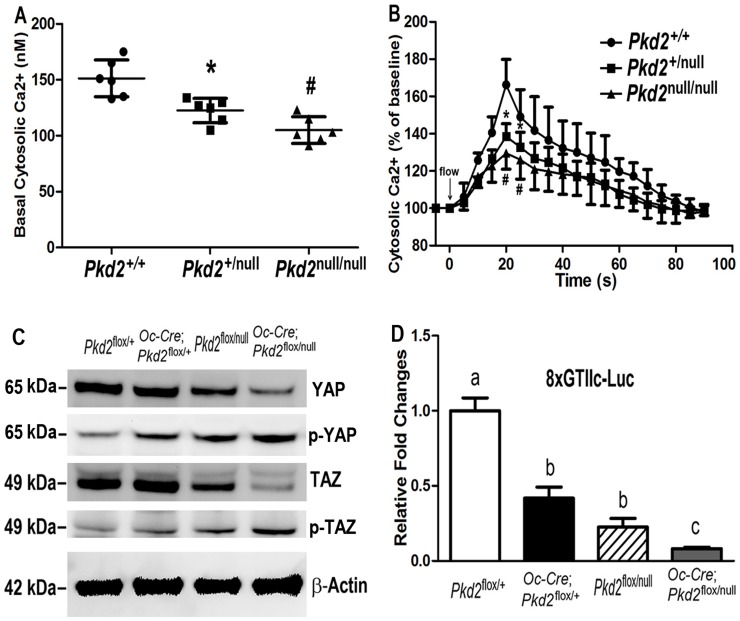
Signaling pathways in *Pkd2* deficiency osteoblasts. (A) Basal intracellular calcium ([Ca^2+^]_i_) levels. Heterozygous *Pkd2* ^+/null^ osteoblasts (n = 6) showed a significantly lower basal [Ca^2+^]_i_ levels compared with wild-type *Pkd2* ^+/+^ osteoblasts (n = 6), and homozygous *Pkd2* ^null/null^ osteoblasts (n = 6) had greater reductions of basal [Ca^2+^]_i_ compared with the heterozygous *Pkd2* ^+/null^ osteoblasts (n = 6). (B) Flow-induced [Ca^2+^]_i_ response. A gene dose-dependent reduction of flow-induced [Ca^2+^]_i_ response was observed in the *Pkd2*-deficient osteoblasts compared with wild-type *Pkd2* ^+/+^ osteoblasts, indicating an impairment of calcium channel activity in the *Pkd2*-deficient osteoblasts. (C) TAZ/YAP-dependent transcriptional activation as assessed by 8xGTIIc-luciferase activity. A gene dose-dependent reduction of basal TAZ/YAP activity was observed the in immortalized primary osteoblasts derived from the *Pkd2-*deficient osteoblasts compared to the wild-type controls. (D) Western blot analysis. Phosphorylation of YAP was significantly increased, while the amount of TAZ was significantly decreased in *Pkd2-*deficient osteoblasts than in wild-type controls. Data are mean±S.D. from triple independent experiments. Values sharing the same superscript in different group are not significantly different at *P*<0.05.

We found that *Pkd2* deficiency had a gene dose effect on basal intracellular calcium ([Ca^2+^]_i_) concentration and flow-induced intracellular calcium response in immortalized *Pkd2*-deficient osteoblasts. In this regard, heterozygous *Pkd2* ^null/+^ osteoblasts showed a significantly lower basal intracellular calcium ([Ca^2+^]_i_) concentration compared with wild type *Pkd2* ^+/+^ cells, and homozygous *Pkd2* ^null/null^ osteoblasts had greater reductions of basal [Ca^2+^]_i_ compared with the heterozygous *Pkd2* ^null/+^ cells (Figure 5A). To study whether PC2-mediated mechanical flow-induced intracellular calcium level changed, these immortalized cells were exposed to 6.24 dynes/cm^2^ pulsatile laminar fluid flow. On fluid stimulation, we detected an immediate rise in intracellular calcium throughout the wild type *Pkd2* ^+/+^ cell population, peaking roughly 10–20 s after stimulation (Figure 5B). The [Ca^2+^]_i_ levels then rapidly decreased but were maintained at moderate levels for 50–60 s before returning to baseline. In contrast, when we exposed these *Pkd2*-deficient osteoblasts to an identical flow stimulus, we detected intermediate calcium response curve in the heterozygous cells and greater reduction of calcium influx in either the peak or late phase in the homozygous osteoblasts (Figure 5B). In addition, 100 nM triptolide stimulated normal calcium influx in wild type *Pkd2* ^+/+^ cells, moderate calcium signals in heterozygous *Pkd2* ^null/+^ cells, and no response in *Pkd2* null cells (data not shown) in the loading chamber, consistent with loss of PC2 abolishes PC2 agonist-induced calcium response in osteoblasts.

To explore mechanisms of decreased *Runx2* and *PPARγ* expression in *Pkd2*-deficient mice, we looked for alterations in the Hippo signaling pathway effectors YAP/TAZ, which play a critical roles in regulating mesenchymal stem cell fate determination into osteoblasts and adipocytes in response to alterations in extracellular matrix rigidity and cell shape [30]. We found that loss of Pkd2 in osteoblasts resulted in significant reduction in *TAZ*, *YAP*, and their transcriptional targets, baculoviral IAP repeat containing 3 (*Birc-3*), connective tissue growth factor (*Ctgf*), and inhibin beta A (*Inhba*) (Table 2). Western blot analysis revealed that phosphorylation of YAP and TAZ were significantly increased (indicating retention of p-YAP and p-TAZ in the cytoplasm) (Figure 5C). We also found that the total amount of YAP and TAZ were significantly decreased in *Pkd2-*deficient osteoblasts (Figure 5C). To examine the effect of *Pkd2* inactivation on TAZ/YAP transcriptional activity, we examined a reporter gene construct with multiple copies of the TEAD-binding GTIIC (GGAATG) site (8xGTIIC-luciferase). YAP and TAZ are coactivators of the transcription factor TEAD, which can be used as a read out of YAP/TAZ activity. GTIIC-luciferase activity was reduced in a *Pkd2* gene dose-dependent manner in immortalized primary osteoblasts derived from the *Pkd2-*deficient osteoblasts compared to the wild-type controls (Figure 5D). These findings indicated that loss of PC2 significantly attenuates both YAP and TAZ components of Hippo signaling pathway in immortalized primary osteoblasts.

In addition, *Sost* is expressed in osteocytes and regulates bone mass through Wnt-dependent signaling. We looked for evidence of activation of Wnt signaling pathway, since *Sost* message expression was decreased in *Pkd2* deficient bone. However, we observed a decrease in *Wnt10b* and *Axin2*, suggesting that loss of Pkd2 resulted in decreased Wnt signaling, in spite of reductions in *Sost*.

## Discussion

Using an *Osteocalcin* (*Oc*) promoter-driven Cre and *Pkd2* ^flox/null^ mouse model, we demonstrate that osteoblast-specific deletion of *Pkd2* results in decreased osteoblast-mediated bone formation and reduction in bone mass *in vivo* and impaired osteoblast differentiation of primary osteoblasts *ex vivo.* In addition, there is decreased bone marrow fat *in vivo* and a reduction of adipogenesis in primary osteoblast cultures derived from *Pkd2*-deficient mice. The reduction in bone mass was due to both loss of trabecular bone volume and diminished cortical thickness and lead to loss of structural integrity as assessed by reduced ability to resist fracture in response to loading *ex vivo*. Reductions in osteoblast-mediated mineral apposition rates and decrements in osteoblast gene expression, as well as the lack of evidence for increased osteoclastic markers in bone or serum, are most consistent with bone loss due to decreased bone formation rather than increased bone resorption. Thus, selective *Pkd2* deficiency causes a low turnover osteopenia.

Consistent with PC1 coupling to PC2, both *Oc*-Cre-mediated reductions of *Pkd1* and *Pkd2* transcripts in osteoblasts cause a low bone mass due to decreased osteoblast-mediated bone formation. However, there are a few notable differences in the bone phenotype of these conditional *Pkd1* and *Pkd2* deficient mice, suggesting that PC1 and PC2 functions are not identical. In this regard, *Pkd1* deficiency in osteoblasts resulted in a more robust gene-dose dependent effects than observed in targeted deletion of *Pkd2* in osteoblasts [12]. A greater effect of PC1 compared to PC2 is also seen in other settings. For example, mutations in *Pkd1* cause a more severe cystic kidney phenotype than mutations in *Pkd2* [31]. In addition, *Pkd1* deficient mice have an inverse effect on osteoblastic and adipocytic differentiation, such that decreased osteoblastic function and osteopenia were associated with a reciprocal enhancement of adipogensis and increased bone marrow fat. In contrast, there was a concordant reduction in osteoblastogenesis and adipogenesis in *Pkd2* deficient mice. These differences might reflect the differential and broader signaling pathways coupled to Pkd1 compared to Pkd2. Indeed, *Pkd1* deficiency resulted in a decrease in *Runx2* and an increase in *PPARγ*, whereas reductions in *Pkd2* resulted in parallel reductions of *Runx2* and *PPARγ*.

We have previously shown that Pkd1 regulation of bone mass is mediated by Runx2 [21]. Compound *Runx2* and *Pkd1* heterozygous mice have additive effects on reduction of bone mass. Also, Pkd1 effects on *Runx2* promoter activity are mediated through coupling to Pkd2 calcium channel activity and regulation of calcium signaling pathways. In this regard, *Pkd1* deficient osteoblasts had lower intracellular calcium and Pkd1 responsive enhancer regions of the *Runx2* promoter were identified in an area containing AP-1 and NFI binding sites. shRNA-mediated reductions in PKD1 in MG-63 osteoblasts also reduced intracellular calcium, attenuated calcium signaling response to shear fluid stress, and increased cAMP responses [27]. It is likely that reduction in *Runx2* message expression in *Pkd2* deficient osteoblasts is related to alterations in similar intracellular calcium signaling. Indeed, osteoblasts derived from *Pkd2*-deficient osteoblasts exhibited lower basal intracellular calcium ([Ca^2+^]_i_) and impaired response to flow-induced intracellular calcium influx, indicating that calcium channel PC2 is coupled to fluid flow sensing PC1 to response to mechanical loading osteoblasts.

**Figure 6:**
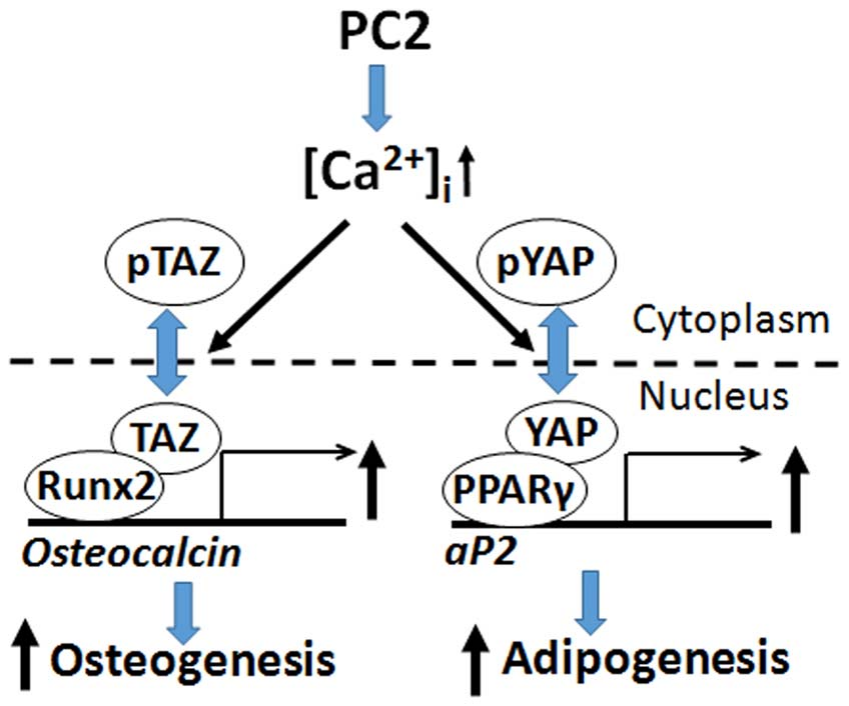
Schema showing potential interactions between polycystins and Hippo signaling pathways in osteoblasts. PC-2 coordinately regulates PPARγ and Runx2 to respectively control adipogenesis and osteoblastogenesis. Hippo signaling effectors Yap and Taz are also coordinately regulated by PC-2 as well as other physical forces that act as co-factors for PPARγ and Runx2. Inverse effects on osteoblast and adipocyte differentiation by PC-1 might be explained by uncoupling PC-1 and PC-2 signaling leading to enhancement of PC-1 C-terminal tail (PC-1-CTT)/Taz signaling and increased Runx2-dependent osteoblastogenesis and decreased PPARγ-mediated adipogenesis.

The differential effects of selective *Pkd1* and *Pkd2* deficiency in osteoblasts on bone marrow adipogenesis suggests that PC1 and PC2 signaling can be uncoupled in bone. This possibility led us to investigate the potential interactions between polycystins and YAP and TAZ, components of the Hippo signaling pathway. TAZ/YAP, like polycystins, are regulated by mechanical and cytoskeletal cues [30], [32]–[36]. In addition, YAP/TAZ differentially regulates mesenchymal precursors toward osteoblastic and adipocytic cell fates [32]. Specifically, TAZ acts as a co-activator of Runx2 and a direct inhibitor of the transcriptional activity of PPARγ [37], [38]. Indeed, TAZ overexpression in osteoblasts stimulates *Runx2* expression, osteoblast differentiation and increases bone mass [39]. YAP, on the other hand, acts as a co-repressor of *Runx2* and co-activator of PPARγ transcriptional activity [40]–[42]. PKA-induced adipogenesis also involves cAMP dependent phosphorylation of YAP [43]. Finally, TAZ binds to PC2 leading to its degradation and to PC1 to both modify its interactions with PC2 and possibly enhance nuclear translocation and transcriptional functions of the PC1 C-terminal tail [44]. We observed reductions in both TAZ and YAP in *Pkd2*-deficient mouse bone. PC-2-depedendent stimulation of TAZ could work in concert with calcium- and PC-1-C-terminal tail dependent stimulation of Runx2 to stimulate osteoblastogenesis; whereas PC-2 stimulation of YAP and promotion of PPARγ activity could stimulate adipogenesis (Figure 6). In this schema, activation of PC-1 results in stimulation of osteoblastogenesis and inhibition of adipogenesis through coordinate effects on Runx2 and TAZ signaling. Further studies are needed to investigate this schema and establish the regulation and functional roles of TAZ and YAP in polycystin control of osteoblast and adipocyte differentiation. Interestingly, the reduction in YAP in *Pkd2* deficient mice is opposite to the up-regulation of YAP in ADPKD [45], suggesting differences in tissue specific regulation of Hippo signaling.

In addition, we observed that both *Fgf23* transcripts in bone and serum Fgf23 levels were decreased in *Pkd2* deficient mice, consistent with the effects of loss of *Pkd2* to suppress osteoblast/osteocyte functions. Recent studies, however, show that that serum FGF23 levels are increased in ADPKD patients [46]–[48] and in a PKD orthologous mouse model [9]. These results imply that the observed FGF23 elevation is due to effects of chronic kidney disease (CKD) to increase FGF23, possibly due to systemic and/or local factors that stimulate FGF23 production or end-organ resistance to FGF23 action in CKD [49]. Alternatively, FGF23 mRNA and protein expression was detected in cell lining renal cysts, but not in bone of the cy/+ Han:SPRD rat model of PKD; similar finding was also observed in an inducible *Pkd1* knockout mouse model, suggesting ectopic expression of FGF23 in CKD [49]. The *Col4a3* null mouse model of CKD also exhibited increased circulating FGF23 levels prior to increased expression of FGF23 in bone [50], [51]. Further studies are needed to understand the mechanism leading to increased FGF23 in CKD, but our studies indicate that specific loss of *Pkd2* in bone results in decreased FGF23 expression, along with other gene products produced by osteoctyes.

In conclusion, osteoblast-targeted deletion of *Pkd2* result in significant bone loss associated with impaired osteoblastic and adipocytic differentiation. Reductions in intracellular calcium and alterations in the YAP/TAZ transcriptional modulators of mesenchymal stem cell differentiation are downstream mediators linking Pkd2 and regulation of Runx2 and PPARγ expression. These findings contrast with prior studies showing that *Pkd1* deficiency results in reciprocal downregulation of Runx2-mediated osteoblastogenesis and upregulation of PPARγ-mediated adipogenesis. Thus, Pkd1 and Pkd2 separately regulate osteogenic and adipogenic pathways. Polycystins in partnership with the Hippo signaling pathway in osteoblasts may have the fundamental function of maintaining the osteoblast differentiation state and by regulating adipocytes differentiation/transdifferentiation in response to environmental factors that include mechanical loading.
